# Comparison of Molecularly Identified Resistant and Susceptible Johnsongrass (*Sorghum halepense* L.) Populations at *ALS* Gene, in the Absence and Presence of Field Crops

**DOI:** 10.3390/genes15111415

**Published:** 2024-10-31

**Authors:** Aristeidis P. Papapanagiotou, Eleni A. Anthimidou, Ilias G. Eleftherohorinos, Ioannis A. Giantsis

**Affiliations:** 1 Department of Agriculture, University of Western Macedonia, 53100 Florina, Greece; apapanagiotou@uowm.gr; 2 School of Agriculture, Aristotle University of Thessaloniki, 54124 Thessaloniki, Greece; anthimidou.lena@gmail.com (E.A.A.); eleftero@agro.auth.gr (I.G.E.)

**Keywords:** spraying, acetolactate synthase gene, crops, weeds, resistance, horticulture, mutation

## Abstract

Background/Objectives: Johnsongrass (*Sorghum halepense*) is an erect tetraploid, perennial, C4 grass weed species categorized among the world’s most noxious weeds due to its high competitive ability against crops and the increased number of field-evolved herbicide-resistant populations. The aim of the present study was to assess the growth rate and performance of resistant (R) johnsongrass genotypes hosting Trp574Leu target-site cross-resistance at *ALS* gene, inhibiting various herbicides, compared to susceptible (S) conspecific weeds, in the absence and presence of corn or sunflower antagonism. Methods: The aboveground biomass, tiller, and rhizome production ability of one S and one R johnsongrass population with a Trp574-Leu substitution conferring cross-resistance to ALS-inhibiting herbicides were compared under non-competitive conditions. Furthermore, the competitive ability of these two johnsongrass populations against corn or sunflower was determined in a target-neighborhood design. Results: The S and R johnsongrass populations displayed similar growth rates concerning aboveground biomass and tiller number, whereas the R population displayed a slightly greater growth rate for rhizome production compared to the S population. Both populations grown with corn produced more aboveground biomass than the ones grown with sunflowers. The aboveground biomass of corn was reduced to a greater extent than sunflower by the presence of both johnsongrass populations, while both crops were affected more by the S than by the R population. Conclusions: Although the inheritance and the genetic background of plant materls were not addressed, the findings of this study indicate clearly that the growth rate and competitive ability of the ALS-resistant johnsongrass population are not associated with the resistance mechanism involved.

## 1. Introduction

Johnsongrass [*Sorghum halepense* (L.) Pers.] is an erect tetraploid (2n = 2x = 40), perennial, C4 grass weed species that reproduces sexually by seeds formed predominately through self- and partially through cross-pollination and asexually by a below-ground rhizome system [1,2]. It originates from the Mediterranean areas of Europe, Africa, and Asia and has gradually invaded new agricultural areas of the world [3].

Johnsongrass is categorized among the world’s most noxious weeds, ranking sixth and infesting 30 crops in more than 50 countries [4,5]. It is responsible for severe infestations in many economically important annual summer crops, such as corn (*Zea mays* L.), sunflower (*Helianthus annuus* L.), soybean [*Glycine max* (L.) Merr.], grain sorghum [*Sorghum bicolor* (L.) Moench], cotton (*Gossypium hirsutum* L.), and vegetables, as well as perennial crops (orchards and vineyards). Johnsongrass is characterized by a remarkable reproductive ability and high biomass accumulation, which enable it to effectively invade, establish, persist, and compete with field crops [6]. The first rhizomes of a plant originating from seed are produced three to six weeks after emergence and when seedlings have four to five leaves [4,7], but the ability of johnsongrass individuals to produce rhizomes depends on the identity of the neighbor-competitor and their origin (agricultural or nonagricultural) [8,9,10]. More specifically, Smith et al. [10] reported that johnsongrass individuals produced more rhizomes when growing in corn than alone or with other johnsongrass populations, whereas the differences in rhizome production between agricultural and nonagricultural johnsongrass populations were modest.

Plants originating from rhizomes exhibit higher growth rates and are more competitive than plants originating from seeds [11,12,13]. This was confirmed by Mitskas et al. [11] who found 88% and 57% corn-grain yield loss associated with season-long interference by 110 to 130 johnsongrass stems m^−2^ from rhizomes or seed, respectively, as compared with the yield from weed-free corn. The greater competitive ability of johnsongrass plants originating from rhizomes than seeds could be attributed to their greater growth rate due to more stored energy reserves that substantially support growth, and possibly due to their capability to house bacterial endophytes that provide beneficial functions related to atmospheric nitrogen fixation [14]. The increased competitive ability of johnsongrass against crops was also documented by Vasilakoglou et al. [15], who found 86% and 41% yield loss of cotton and corn, respectively, due to season-long interference by 100 to 200 johnsongrass stems m^−2^, originating from planted rhizomes.

Post-emergence applications with acetolactate synthase (ALS)-inhibiting herbicides foramsulfuron, nicosulfuron, and rimsulfuron are commonly used for the control of johnsongrass infestation occurring from seed and overwintering rhizomes [16,17]. As the ALS enzyme is by far the most resistance-prone site of herbicidal action, the intensive use of these herbicides has been proposed to impose a strong selective pressure that has led to the evolution of 174 resistant weed species globally [18]. Since many of these resistant weed species infest important food crops, their evolution threatens food security for the increasing world population. Johnsongrass is one of these species, with several field-selected populations having evolved cross-resistance to either ALS- [19,20,21] or ACCase-inhibiting herbicides [22,23,24,25], as well as resistance to 5-enolpyruvylshikimate-3-phosphate synthase (EPSPS) [6] or multiple resistance to both EPSPS and ACCase-inhibiting herbicides [18].

Weed fitness refers to their capacity to survive and reproduce, while fitness cost or adaptation cost of resistant weeds refers to the reduction of this capacity in the absence of a herbicide selective pressure, and is associated with negative pleiotropic effects of the resistance-endowing mutant allele(s) [26,27]. According to Cousens and Fournier-Level [28], Keshtkar et al. [29], and Vila-Aiub [30], fitness cost is manifested through changes in plant traits such as seed size, seed yield per plant, seed dormancy, seed germination rate, establishment, survival, early vigor, phenology, pollination, biomass (aboveground and rhizome) production, and competitive ability that affect the frequency of resistant plants as compared with that of plants not harboring resistance alleles. Moreover, the fitness of resistant weeds has been shown to depend on several factors, such as plant species, resistance mechanism(s) involved, specific mutant resistance allele(s), genetic background, genetic diversity, gene flow, and gene drift, pleiotropic effects on the kinetics of herbicide target proteins, natural selection, agricultural practices, abiotic and biotic environmental conditions [30,31].

Regarding the ecological evolutionary context of fitness, the expected outcome in the absence of herbicide is that the susceptible individuals will eventually dominate the field because they are more fit compared to the herbicide-resistant individuals [29,32]. On the other hand, in the presence of a herbicide, the herbicide-resistant individuals, regardless of the fitness cost, will eventually dominate the field due to removal–control of the susceptible ones, eliminated by repeated herbicide applications. In addition, if the gene mutation confers a significant level of resistance and results in neutral [26] or positive fitness [29], it will eventually lead to the rapid evolution and establishment of herbicide resistance.

Our study was prompted by the genetic and phenotypic variation reported between johnsongrass populations [8,9,10], along with the generally expected inconsistent fitness differences between herbicide-resistant and herbicide-susceptible weed populations [24,33]. Therefore, the objectives of this study were (a) to assess the growth rate (aboveground biomass, tiller number, and rhizome production) of one R population with a Trp574Leu target-site cross-resistance to ALS-inhibiting herbicides and one S population in the absence of crop competition, and (b) to investigate their competitive ability in the presence of corn or sunflower plants.

## 2. Materials and Methods

### 2.1. Seed Collections and Molecular Characterization

The ALS-R johnsongrass population seeds were collected from plants originating from a corn monoculture field repeatedly treated with the maximum label field rate of post-applied sulfonylurea herbicides, located in the prefecture of Kavala, northeastern Macedonia, Greece (40°55′26″ N 24°37′17″ E). A representative sample of seeds was collected by hand from approximately 70 to 80 individual johnsongrass plants that survived the ALS-inhibitor herbicides applied in corn. Seeds were pooled together and were characterized as a putative-resistant population and their field-evolved resistance was verified in subsequent whole-plant rate–response assays conducted in pot experiments [21]. The S johnsongrass population seeds were collected from plants grown in another location in northern Greece (Aristotle University farm, Thessaloniki, Central Macedonia, Greece, 40°31′13″ N, 22°58′18″ E), which had no history of exposure to either ALS- or ACCase-inhibiting herbicides (these seeds were considered as the S population).

It should be additionally stressed that, despite the approximately 150–200 km distance of these two populations, their genetic background was minimized by applying the “recurrent pedigreed lines” methodology, i.e., intermating and selfing the isolated populations after collecting and labeling plant families (see details in [29]) in an effort to increase the homogeneity of the genetic background of these weed populations.

For confirmation of the presence of the resistance-endowing mutation in the R population and its absence in the wild-type S population, molecular identification was conducted in a total of 40 representative randomly sampled seeds, by PCR and sequencing. Briefly, after DNA extraction using the NucleoSpin Plant tissue kit (Macherey-Nagel, Duren, Germany), the KAPA2G Fast HotStart PCR Kit (KAPABIOSYSTEMS, Wilmington, MA, USA) was utilized to amplify a 1364 base pairs segment of the *ALS* gene, using the primers ECH-5F and ECH-3R, with volumes and PCR conditions as described in [21]. Amplicons were purified and sequences in an ABI-PRISM 3730 automatic sequencer (Applied Biosystems, Foster City, CA, USA).

### 2.2. Investigation of Growth Rate

The aboveground biomass, tiller, and rhizome production ability of one S and one ALS-R johnsongrass population with target-site mediated broad cross-resistance to ALS-inhibiting herbicides foramsulfuron, nicosulfuron, rimsulfuron, and imazamox due to a Trp574-Leu substitution were compared under non-competitive conditions. The field-selected johnsongrass population was evaluated in the growth rate and the competition study against important field crops as it displayed high broad cross-resistance to various ALS-inhibiting herbicides [the calculated GR_50_ value (herbicide rate (g ai ha^−1^) required for 50% reduction of its fresh weight) was estimated to be 295, 75, and 104 g ai ha^−1^ for the sulfonylurea herbicides foramsulfuron, nicosulfuron, and rimsulfuron, respectively] [21]. The choice of a reference population that had never been exposed to herbicide applications allowed a significant comparison between a highly ALS-resistant and a highly susceptible johnsongrass population in vegetative and reproductive plant traits and their competitive ability against field-row crops.

The experiment was conducted at the farm of Aristotle University of Thessaloniki from June to September 2019, using plastic pots (20 by 25 by 30 cm). The pots were filled with soil having 32% clay, 56% silt, 12% sand, 1.5% organic matter, 7.5% CaCO_3_, pH 7.7 (1:1 H_2_O), and 28.6 meq 100 g^−1^ cation exchange capacity. Uniform-sized seeds of the R and S johnsongrass populations were pre-germinated before their use in the growth rate and weed/crop suppression experiments. In particular, the johnsongrass seeds were initially exposed to concentrated H_2_SO_4_ for approximately 4 to 5 min and subsequently were immersed in a 1.5% solution of KNO_3_ for 2 h. After germination, johnsongrass seedlings with approximately 1 cm shoot length were transplanted into small jiffy pots and allowed to grow until they reached the one-leaf stage. Then, uniform johnsongrass seedlings at the one-leaf stage of the R or S population were transplanted into the center of each pot during June 2019 and allowed to grow up to September. The individual plants of the R population grown in jiffy pots, before their transplanting, were not treated with the labeled rate of foramsulfuron to eliminate possible individual susceptible plants because our previous study [21] had indicated that all plants of this population had survived after the application of the foramsulfuron labeled rate, while those of the S population were completely controlled. All pots were placed outdoors, where they were irrigated and fertilized to maintain vigorous growth throughout the duration of the experiment. Emerging grass and broad-leaf weeds were carefully removed manually throughout the experiment to ensure the absence of other weed competition. The experiment was repeated twice using a completely randomized design with three replications for each sampling time treatment. Plant growth of the R or the S johnsongrass plants was evaluated following seven consecutive destructive samplings, which were performed 5, 6, 7, 8, 9, 10, and 11 weeks after transplanting. For the comparison of the growth rate of the R and S johnsongrass populations, the aboveground biomass (fresh weight) of the plants cut above the soil surface, the tiller number, and the rhizome fresh weight were determined and subsequently used for the analysis of variance (ANOVA). The data obtained were analyzed separately for each of the two investigated crops but were combined over the two runs. As the ANOVA indicated no significant differences between the experiments [the homogeneity of variances checked by Bartlett’s test [34] indicated no significant departure of normality], the data of johnsongrass parameters determined, pooled over the two runs, were regressed against sampling times. In these regression equations, the aboveground biomass, tiller number, and rhizome fresh weight of johnsongrass were the dependent variables (y) and the sampling time was the independent variable (x). Differences between the calculated slope coefficients for R and S populations were compared using the *t*-test at *p* = 0.05.

### 2.3. Johnsongrass Competition with Corn or Sunflower

**Figure 1:**
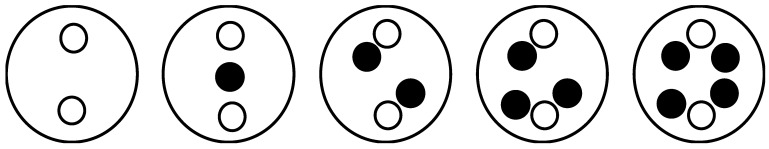
Schematic presentation of the crop/weed density pattern (2:0, 2:1, 2:2, 2:3, 2:4) to assess plant responses of corn or sunflower grown in pure stands and in competition with the R (ALS-herbicide resistant) or S (sensitive) johnsongrass populations [corn or sunflower plants = open circles vs. R or S johnsongrass plants = black circles].

A target-neighborhood design was conducted to evaluate the competitive ability of R and S johnsongrass populations against corn or sunflower. More specifically, the aboveground biomass and tiller number production ability of these two populations were determined under competitive conditions in plastic pots (20 by 25 by 30 cm) with corn (Hamilton hybrid seed, American Genetics Ltd., Thessaloniki, Greece) or sunflower (Neoma hybrid, Syngenta Hellas Seeds, Athens, Greece). The two runs of the experiment were conducted at the farm of Aristotle University of Thessaloniki from June to September 2019 (the same time as that of the growth rate experiment). The pots were filled with soil having the characteristics described above in the growth rate pot experiment. A template was used to achieve an identical distance between each neighbor plant and the target plant, and a uniform distance among neighbor plants (Figure 1). Each pot for the johnsongrass/crop (corn or sunflower) competition experiment was seeded with two crop seeds placed in two hills, spaced 17 cm apart. There were three crop-seeded pots with two seeds per hill spaced 17 cm, which were used as a weed-free crop control. The other treatments consisted of two corn or two sunflower seeds per hill with one, two, three, and four johnsongrass plants transplanted at the one-leaf stage, as shown in Figure 1. Transplanting was performed when corn plants reached the one-leaf stage and sunflower plants reached the cotyledon stage and was followed by the careful thinning to one crop plant per hill in each pot. All pots were placed outdoors and were irrigated and fertilized to maintain vigorous growth throughout the experiment. Emerging grass and broad-leaf weeds were manually removed throughout the duration of the experiment to ensure the absence of competition arising from the presence of other weeds. The establishment of the experiments and their duration were similar to those conducted for the growth rate study (June to September 2019).

Plant growth was evaluated at harvest (September 2019) by determining the aboveground biomass (by cutting plants at the soil surface) of the two corn or sunflower plants, along with the aboveground biomass and tiller number of the one, two, three, and four johnsongrass plants per pot. Each experiment (johnsongrass grown in competition with corn or sunflower) was conducted twice, using a completely randomized design with three replications. The data obtained were analyzed separately for each crop but combined over the two runs. As the ANOVA indicated no significant differences between the experiments [the homogeneity of variances checked by Bartlett’s test [34] indicated no significant departure of normality], the data of either crop or johnsongrass growth traits determined, pooled over the two runs, were regressed against weed density applying lack-of-fit tests. In these regression equations, the aboveground biomass of either crop plants or the aboveground biomass or tiller number of johnsongrass were the dependent variables (y), while the weed density was the independent variable (x). Differences between the calculated slope coefficients for R and S populations were compared using the *t*-test at *p* = 0.05 in SPSS Statistics version 22.0 package.

## 3. Results

### 3.1. Molecular Confirmation of Mutations

All R individuals analyzed were homozygous at the Trp574Leu (TTG) mutation, whereas all S individuals were homozygous at the Trp-574 (TGG) codon site. No double peak was detected that would indicate a heterozygous genotype.

### 3.2. Growth Rate Results

**Figure 2:**
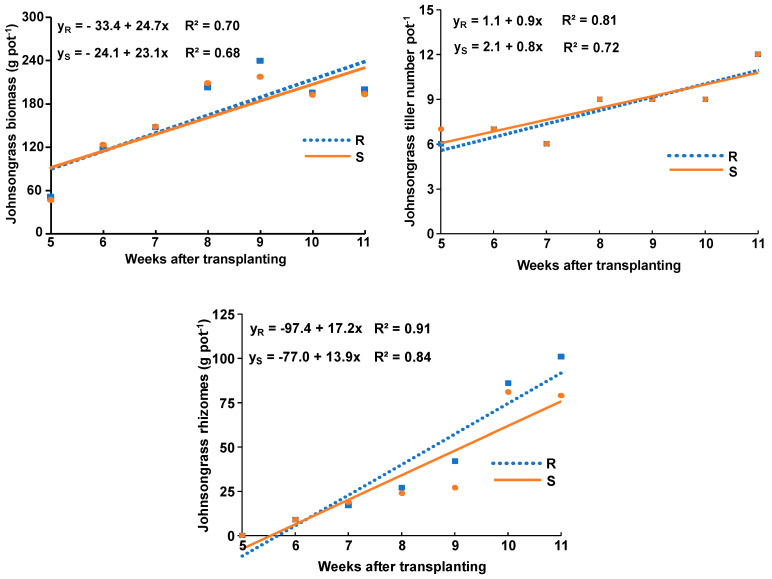
Linear equation and coefficient of determination of aboveground biomass, number of tillers and rhizomes produced by the R and S Johnsongrass populations grown in the absence of crop competition and monitored throughout the life cycle by seven destructive samplings.

The linear equation was the best fit for the regression performed between aboveground biomass, tiller numbers, and rhizome weight against the sampling time of each population. The calculated slopes of linear equations for the aboveground biomass of R and S populations were similar (24.7 ± 0.1 and 23.1 ± 0.1, respectively), and this was also the case for the respective slopes of tiller number (0.8 ± 0.02 and 0.9 ± 0.02). By contrast, the slope for the rhizomes produced by the R population was slightly greater (17.2 ± 0.3) than that of the S johnsongrass population (13.9), but this difference, according to the *t*-test, is not statistically significant (Figure 2).

### 3.3. Johnsongrass Competition with Corn or Sunflower

**Figure 3:**
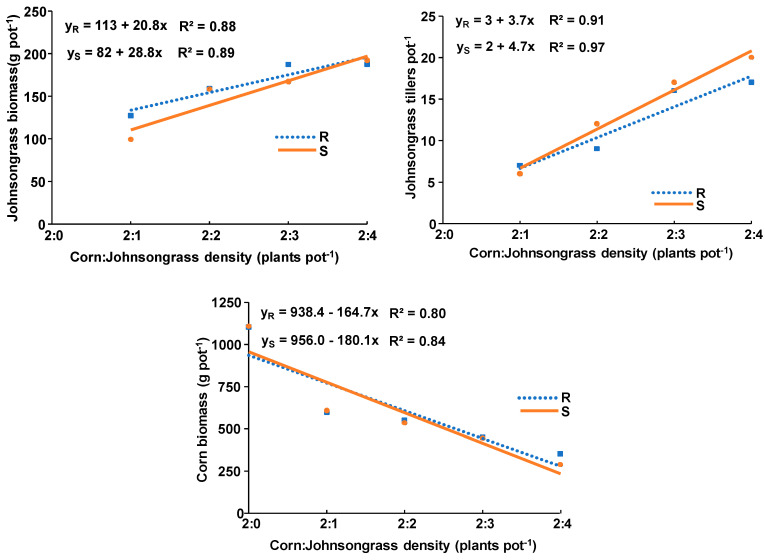
Linear equation and coefficient of determination of aboveground biomass and number of tillers produced by the R and S Johnsongrass populations grown in competition with corn, as well as of the aboveground corn biomass against weed density.

The linear equation, as with the data determined in the growth rate experiment, was again the best fit for the regression performed between aboveground biomass and tiller number against the density of each johnsongrass population grown in competition with corn. The calculated slopes for the aboveground biomass of R and S johnsongrass populations were 20.8 and 28.8, while the respective ones for tiller number were 3.7 and 4.7 (Figure 3). Although the aboveground biomass and tiller number slopes of the R population were slightly lower than those of the S population, these differences were not statistically significant according to the *t*-test.

Corn plants exposed to competition of one to four plants of the R and S johnsongrass populations displayed 46 to 68% and 45 to 74% reduction in aboveground biomass, respectively, as compared with weed-free corn (Figure 3). The calculated slopes for the aboveground biomass reduction of corn due to the presence of R or S populations were −164.7 and −180.1. Again, although corn was affected more by the S than by the R johnsongrass population, this difference was not statistically significant.

**Figure 4:**
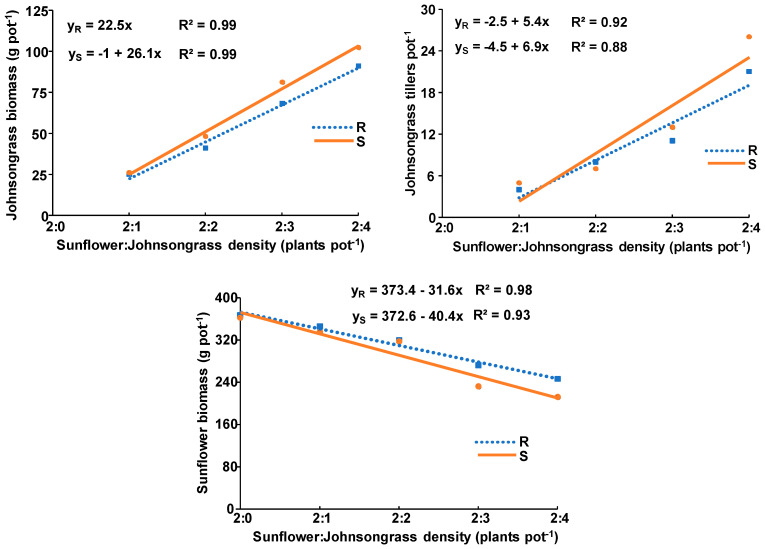
Linear equation and coefficient of determination of aboveground biomass and number of tillers produced by the R and S Johnsongrass populations grown in competition with sunflower, as well as the aboveground sunflower biomass against weed density.

The calculated slopes for the aboveground biomass of the R and S johnsongrass populations grown in competition with sunflower were 22.5 and 26.8, respectively, whereas the respective slopes for tiller number were 5.4 and 6.9 (Figure 4). As above, with corn, the recorded weed differences were not statistically significant.

The calculated slopes for the aboveground biomass of sunflower grown in competition with the R and S johnsongrass populations were −31.6 and −40.4, respectively (Figure 4). As previously, although the R population has a lower competitive ability against sunflower than the S population, this difference was not statistically significant.

Although the ANOVA for johnsongrass populations grown in competition with corn or sunflower was performed separately for each crop, the comparison of the results obtained indicates that the aboveground biomass of both johnsongrass populations grown in competition with sunflower was lower than with corn (Figure 3 and Figure 4). In particular, the aboveground biomass of one, two, three, and four plants of the R johnsongrass population grown in competition with sunflower was 80%, 74%, 64%, and 51% lower than grown in competition with corn, whereas the respective reduction due to the presence of the S johnsongrass population was 74%, 70%, 51%, and 47%.

## 4. Discussion

The slightly higher estimated slope for the rhizomes produced by the R population than that of the S johnsongrass population strongly suggests that the R population carrying the Trp574Leu mutant allele exhibited a greater growth rate for rhizome production than the S population (Figure 2), which increases its ability to spread and establish more effectively, and, in some cases, to be more aggressive against neighbor plants of other weed species or crops. A greater growth rate for rhizome production by resistant johnsongrass populations was also reported by Panozzo and Sattin [24], who found that the R johnsongrass populations from Italy, with resistance to ACCase-inhibiting herbicides due to the target-site mutation Ile-Asn in position 2041 of the *ACCase* gene, allocated 12% more biomass to rhizomes than the S populations when grown in the absence of crop competition. The similar aboveground biomass and tiller number produced by both R and S populations are in contrast with the results of Panozzo and Sattin [24], who found that the ACCase-resistant johnsongrass population due to the Ile2041Asn substitution of the ACCase enzyme allocated 13% and 30% less aboveground and panicle biomass, respectively, than the S population. Ntoanidou et al. [35] also found different growth rates between wild mustard (*Sinapis arvensis* L.) populations, which were related to the geographical origin and the herbicide resistance profile of field-selected populations (Trp574-Leu, Leu574-Leu). However, Yu et al. [36] reported that ALS-herbicide-resistant annual ryegrass (*Lolium rigidum* Gaudin) plants homozygous for this specific ALS resistance substitution (Leu574Leu) had no major impact on ALS functionality, plant growth and probably no plant resistance costs. These fitness differences between R and S populations, according to Vila-Aiub et al. [32], Cousens and Fournier-Level [28], Keshtkar et al. [29], and Vila-Aiub [30], could be attributed to plant changes due to the underlying resistance mechanism, but they are also related to seed production, seed dormancy, seed dispersal, seed longevity, seed germination pattern, early vigor, growth rate, establishment, survival, phenology, pollination, and biomass production and allocation, competitive ability, and response to plant pathogens.

The similarly calculated slopes for the aboveground biomass and tiller number of S and R johnsongrass populations grown in competition with corn or sunflower suggest that their competitive ability is not associated with herbicide resistance. In addition, the similarly calculated slopes for the aboveground biomass reduction of corn or sunflower due to the presence of R or S populations could be attributed to their similar competitive ability against both crops.

The fact that the aboveground biomass of both johnsongrass populations grown in competition with sunflower was lower than with corn supports the evidence of fitness dependence on crop species. The lower reduction of sunflower biomass due to competition of both johnsongrass populations as compared with that of corn could be attributed to the reduced weed interference ability as a result of the greater root system of sunflower that improved nutrient use efficiency and thus provided greater competitive ability and possibly sunflower’s allelopathic capacity. This is confirmed by the lower aboveground biomass of both johnsongrass populations grown in competition with sunflower as compared with those grown with corn. Similar results were reported by Vercellino et al. [37] who found a 12% yield loss of sunflower compared to 100% and 74% of wheat or oilseed rape, exposed under field conditions to season-long interference with ALS-resistant feral radish (*Raphanus sativus* L.) populations. The lower yield reduction of sunflower could result from the lower biomass (115 g m^−2^) produced by the feral radish plants grown in competition with sunflower than with that (1050 g m^−2^) grown with oilseed rape or wheat.

The comparison of the results obtained at the last sampling from one johnsongrass plant grown in the absence of crop competition (growth rate experiment) with those determined for one johnsongrass plant grown in competition with two crop plants (either corn or sunflower) indicates that aboveground biomass of both johnsongrass populations was significantly lower when grown with corn or sunflower than grown alone. More specifically, the aboveground biomass of one R or S johnsongrass plant grown in competition with sunflower was 87% and 87% lower than when grown alone (Figure 2, Figure 3 and Figure 4), whereas the respective reduction in the case of corn presence was 36% and 49% lower than grown alone. The lower aboveground biomass of one plant of R or S johnsongrass populations grown with two sunflower or corn plants as compared with one plant grown in the absence of crop competition was expected, due to suppression impacted by the crops. The fact that the growth of one R or S johnsongrass plant grown in competition with sunflower was affected differently as compared with that grown alone could be attributed to different competitive abilities between weed populations and crop species. In contrast to these results, Smith et al. [10] found that johnsongrass individuals in fertilized pots produced similar aboveground biomass when grown alone or in competition with corn. However, the similar aboveground biomass and tiller number growth rates of both R and S johnsongrass populations grown in the absence of competition, as compared with their differences when grown in competition with corn or sunflower, suggests the necessity of studying growth rates and the fitness of R and S weed populations under both the absence and presence of crop interference, particularly in the presence of crop where weed resistance has evolved.

The lack of statistically significant difference between the growth rates calculated for the R and S populations grown in the presence of either corn or sunflower is in contrast with the results reported by Zhao et al.’s [38] study on the shortawn foxtail (*Alopecurus aequalis* Sobol.) populations grown in competition with wheat. More specifically, they found that the ALS-resistant shortawn foxtail plants, carrying a homozygous *ALS* gene mutation resulting in the Leu574Leu amino acid substitution and grown in competition with wheat, exhibited stronger competitive responses for resources compared with ALS wild-type plants. These contradictory findings show that the fitness of a herbicide-resistant population, which finally alters its competitive ability with other weed populations and crop plants, could not be attributed only to pleiotropic effects caused by the Leu574 mutation, but that the possible existence of linkage between the resistance gene(s) and other unknown gene(s) or phenotypic correlations with other traits could account for this [28,29,30,33]. Therefore, fitness cost expressed due to field-evolved mutant alleles endowing ALS target-site herbicide resistance is difficult to predict [28,32,39], because it may result from insufficient product biosynthesis due to changes in enzyme functionality expressed as impaired enzyme activity, reduced substrate affinity, or altered feedback inhibition [39].

The findings of this study indicate clearly that the growth rate and competitive ability of the ALS-resistant johnsongrass population are not associated with the resistance mechanism involved. Similarly, the associated detrimental effects on ALS functionality and plant growth were not determined in the ALS-resistant rigid ryegrass (*Lolium rigidum* Gaud.) [36] and wild radish (*Raphanus raphanistrum* L.) [40] hosting the Leu574 mutant allele. Moreover, significant differences concerning growth and competitiveness between S and ALS-R redroot pigweed (*Amaranthus retroflexus* L.) populations harboring the Leu574 mutant allele were not determined [41]. In contrast, the Trp574Leu amino acid substitution in field-selected ALS-R annual bluegrass (*Poa annua* L.) was associated with decreased fitness traits (reduced seed yield, tillering, and flowering time) [42]. Also, an ecological fitness penalty (reduced aboveground biomass, reproductive productivity, and plant yield) was determined in ALS-R feral radish (*R. sativus* L.) with the Trp574Leu substitution when grown in competition with wheat [43]. Moreover, the specific mutant resistance-conferring allele, the genetic background, the possible pleiotropic effects on the kinetics of herbicide target proteins, crop species grown in competition, the origin of populations, abiotic and biotic environmental conditions, and agricultural practices could account for this [33].

The more rhizomes produced by the R than the S population in the absence of competition can have an impact on resistance management since the plants of the R populations originating from seeds could be controlled by the application of preemergence herbicides, whereas those originating from rhizomes are not controlled either by the ALS-post-emergence herbicides (due to evolution of resistance) or by preemergence herbicides (due to the lack of effective herbicides against rhizomes) [24]. Therefore, since there are no alternative chemical options available in the market to effectively control johnsongrass in corn other than ALS-inhibiting herbicides, the management of field-evolved ALS-resistant johnsongrass populations becomes very difficult. For this reason, the implementation of crop rotation with either crop having different life cycles (winter cereals and pulses), the more competitive sunflower or other broad-leaved crops that would also allow the use of herbicides with a different mode of action (ACCase inhibitors) to provide effective chemical control of field-selected ALS-resistant johnsongrass populations and reduce the risk of evolution of new resistant populations [21]. Moreover, the integration of complementary methods [37] such as in-season herbicide resistance monitoring for early detection of putative-resistant patches of johnsongrass in corn fields, practicing appropriate soil tillage and stale seedbed preparation, adjusting planting dates, choosing more competitive cultivars, applying nonselective herbicides pre-sowing, carrying out appropriate placement of fertilizers and irrigation management, preventing field-to-field dispersal of johnsongrass rhizomes and seeds, can mitigate herbicide resistance evolution.

The most important limitation of our work is the lack of inheritance and genetic background of the resistance mechanism that strongly affects its evolutionary implications. However, taking into consideration that eight times higher than the field recommended rate of the herbicides foramsulfuron, imazamox, and rimsulfuron were required for the effective control of the determined resistant plants in the field-selected ALS-R population as heterozygous (Trp574Leu) [21], it could be suggested that the resistant *ALS* single-gene acts in an additive-to-dominant nature. Therefore, as johnsongrass is a self- and partially cross-pollinated, C4, tetraploid, and perennial species that reproduces sexually by seeds and asexually by a below-ground rhizome system, along with the possible action of the resistant *ALS* single-gene in an additive-to-dominant nature, the evolution of herbicide resistance can occur rapidly because it can be selected even in heterozygous plants and can spread effectively by both seed and pollen. In addition, although johnsongrass is a tetraploid species harboring two different subgenomes, the herbicide resistance on the ploidy level has not been examined yet [21] and, for this reason, its evolutionary implications are impossible to predict.

## 5. Conclusions

The results of this study allow the following conclusions to be drawn:

(1) The S and ALS-cross R johnsongrass populations displayed similar growth rates concerning aboveground biomass and tiller number, whereas the R population displayed a slightly greater growth rate for rhizome production compared to the S population.
(2) Both populations grown with corn produced more aboveground biomass than the ones grown with sunflowers.
(3) The aboveground biomass of corn was reduced to a greater extent than sunflower by the presence of both johnsongrass populations, while both crops were affected more by the S than by the R population.
(4) The growth rate and competitive ability of the ALS-resistant johnsongrass population are not associated with the resistance mechanism involved, suggesting that other factors are more important for the differentiation of johnsongrass growth traits.

Based on the strongly supported lack of association between plant fitness (growth rate and competitive ability) and the ALS cross-resistance of the johnsongrass population, future research should be focused on the inheritance and genetic background of the resistance mechanism that strongly affects fitness and its evolutionary implications, and also on the development and implementation of alternative to chemical control methods and agronomic practices [44].

## Data Availability

The original contributions presented in this study are included in the article, and further inquiries can be directed to the corresponding authors.

## References

[1] Warwick S.L., Black L.D. (1983). The biology of Canadian weeds. 61. *Sorghum halepense* (L.) Pers. Can. J. Plant Sci..

[2] Fernandez L., de Haro L.A., Distefano A.J., Martinez M.C., Lia V., Papa J.C., Olea L., Tosto D., Hopp H.E. (2013). Population genetics structure of glyphosate-resistant Johnsongrass (*Sorghum halepense* L. Pers) does not support a single origin of the resistance. Ecol. Evol..

[3] Follak S., Essl F. (2013). Spread dynamics and agricultural impact of *Sorghum halepense*, an emerging invasive species in Central Europe. Weed Res..

[4] Holm L.G., Plucknett D.L., Pancho J.V., Herberger J.P. (1977). The World’s Worst Weeds: Distribution and Biology.

[5] Peerzada A.M., Ali H.H., Hanif Z., Bajwa A.A., Kebaso L., Frimpong D., Iqbal N., Namubiru H., Hashim S., Rasool G. (2017). Eco-biology, impact, and management of *Sorghum halepense* (L.) Pers. Biol. Invasions.

[6] Riar D.S., Norsworthy J.K., Johnson D.B., Scott R.C., Bagavathiannan M. (2011). 2011. Glyphosate resistance in a Johnsongrass (*Sorghum halepense*) biotype from Arkansas. Weed Sci..

[7] Paterson A.H., Kong W.Q., Johnston R.M., Nabukalu P., Wu G., Poehlman W.L., Goff V.H., Isaacs K., Lee T.-H., Guo H. (2020). The evolution of an invasive plant, *Sorghum halepense* L. (‘Johnsongrass’). Front Genet..

[8] Atwater D.Z., Sezen U.U., Goff V., Goff V., Kong W., Paterson A.H., Barney J.N. (2015). Reconstructing changes in the genotype, phenotype, and climatic niche of an introduced species. Ecography.

[9] Atwater D.Z., Fletcher R.A., Dickinson C.C., Paterson A.H., Barney J.N. (2018). Evidence for fine-scale habitat specialisation in an invasive weed. J. Plant Ecol..

[10] Smith A.L., Atwater D.Z., Kim W., Haak D.C., Barney J.N. (2021). Invasive plant rhizome production and competitiveness vary based on neighbor identity. J. Plant Ecol..

[11] Mitskas B.M., Tsolis C.E., Eleftherohorinos I.G., Damalas C.A. (2003). Interference between corn and Johnsongrass (*Sorghum halepense*) from seed or rhizomes. Weed Sci..

[12] Acciaresi H.A., Guiamet J.J. (2010). Below- and above-ground growth and biomass allocation in maize and *Sorghum halepense* in response to soil water competition. Weed Res..

[13] Karkanis A., Athanasiadou D., Giannoulis K., Karanasou K., Zografos S., Souipas S., Bartzialis D., Danalatos N. (2020). Johnsongrass (*Sorghum halepense* (L.) Pers) interference, control and recovery under different management practices and its effects on the grain yield and quality of maize crop. Agronomy.

[14] Rout M.E., Chrzanowski T.H. (2009). The invasive *Sorghum halepense* harbors endophytic N2-fixing bacteria and alters soil biogeochemistry. Plant Soil.

[15] Vasilakoglou I., Dhima K., Eleftherohorinos I. (2005). Allelopathic potential of bermudagrass and Johnsongrass and their interference with cotton and corn. Agron. J..

[16] Eleftherohorinos I.G., Kotoula-Syka E. (1995). Influence of herbicide application rate and timings for post-emergence control of *Sorghum halepense* (L.) Pers. in maize. Weed Res..

[17] Travlos I.S., Montuli J.M., Kukorelli G., Malidza G., Dogan M.N., Cheimona N., Antonopoulos N., Kanatas P.J., Zannopoulos S., Peteinatos G. (2019). Key aspects on the biology, ecology and impacts of Johnsongrass [*Sorghum halepense* (L.) Pers] and the role of glyphosate and non-chemical alternative practices for the management of this weed in Europe. Agronomy.

[18] Heap I. The International Survey of Herbicide Resistant Weeds. http://www.weedscience.org.

[19] Panozzo S., Milani A., Scarabel L., Balogh A., Dancza I., Sattin M. (2017). Occurrence of different resistance mechanisms to ALS inhibitors in European *Sorghum halepenese*. J. Agric. Food Chem..

[20] Werle R., Begcy K., Yerka M.K., Mower J.P., Dweikat I., Jhala A.J., Lindquist J.L. (2017). Independent evolution of acetolactate synthase-inhibiting herbicide resistance in weedy *Sorghum* populations across common geographic regions. Weed Sci..

[21] Papapanagiotou A.P., Loukovitis D., Ntoanidou S., Eleftherohorinos I.G. (2022). Target-site cross-resistance to ALS-inhibitors in Johnsongrass originating from Greek cornfields. Weed Technol..

[22] Kaloumenos N.S., Eleftherohorinos I.G. (2009). Identification of a Johnsongrass (*Sorghum halepense*) biotype resistant to ACCase-inhibiting herbicide in northern Greece. Weed Technol..

[23] Scarabel L., Panozzo S., Savoia W., Sattin M. (2014). Target-site ACCase-resistant Johnsongrass (*Sorghum halepense*) selected in summer dicot crops. Weed Technol..

[24] Panozzo S., Sattin M. (2021). Fitness costs associated to an Ile2041Asn mutation in the geophyte *Sorghum halepense* resistant to ACCase-inhibiting herbicides. Front Agron..

[25] Papapanagiotou A.P., Loukovitis D., Damalas C.A., Eleftherohorinos I.G. (2022). Identification of an ACCase-resistant Johnsongrass (*Sorghum halepense* L.) population from a cotton field in northern Greece. Weed Biol. Manag..

[26] Vila-Aiub M.M., Neve P., Roux F. (2011). A unified approach to the estimation and interpretation of resistance costs in plants. Heredity.

[27] Vila-Aiub M.M., Gundel P.E., Preston C. (2015). Experimental methods for estimation of plant fitness costs associated with herbicide-resistance genes. Weed Sci..

[28] Cousens R.D., Fournier-Level A. (2018). Herbicide resistance costs: What are we actually measuring and why?. Pest Manag. Sci..

[29] Keshtkar E., Abdolshahi R., Sasanfar H., Zand E., Beffa R., Dayan F.E., Kudsk P. (2019). Assessing fitness costs from a herbicide-resistance management perspective: A review and insight. Weed Sci..

[30] Vila-Aiub M.M. (2019). Fitness of herbicide-resistant weeds: Current knowledge and implications for management. Plants.

[31] Yanniccari M., Vila-Aiub M., Istilart C., Acciaresi H., Castro A. (2016). Glyphosate resistance in perennial ryegrass (*Lolium perenne* L.) is associated with a fitness penalty. Weed Sci..

[32] Vila-Aiub M.M., Neve P., Powles S.B. (2009). Fitness costs associated with evolved herbicide resistance alleles in plants. New Phytol..

[33] Baucom R. (2019). Evolutionary and ecological insights from herbicide-resistant weeds: What have we learned about plant adaptation and what is left to uncover?. New Phytol..

[34] Snedecor G.W., Cochran W.G. (1989). Statistical Methods.

[35] Ntoanidou S., Madesis P., Menexes G., Eleftherohorinos I. (2020). Growth rate and genetic structure of *Sinapis arvensis* susceptible and herbicide resistant populations originating from Greece. Euphytica.

[36] Yu Q., Han H., Vila-Aiub M.M., Powles S.B. (2010). AHAS herbicide resistance endowing mutations: Effect on AHAS functionality and plant growth. J. Exp. Bot..

[37] Vercellino R.B., Pandolfo C.E., Cantamutto M., Presotto A. (2024). Interference of feral radish (*Raphanus sativus*) resistant to AHAS-inhibiting herbicides with oilseed rape, wheat, and sunflower. Intern. J. Pest Manag..

[38] Zhao N., Yan Y., Du L., Zhang X., Liu W., Wang J. (2020). Unravelling the effect of two herbicide resistance mutations on acetolactate synthase kinetics and growth traits. J. Exp. Bot..

[39] Yu Q., Powles S.B. (2014). Resistance to AHAS inhibitor herbicides: Current understanding. Pest Manag. Sci..

[40] Li M., Yu Q., Han H., Vila-Aiub M., Powles S.B. (2013). ALS herbicide resistance mutations in *Raphanus raphanistrum*: Evaluation of pleiotropic effects on vegetative growth and ALS activity. Pest Manag. Sci..

[41] Wang R., Han Y., Sun Y., Huang H., Wei S., Huang Z. (2022). Growth and competitiveness of ALS-inhibiting herbicide-resistant *Amaranthus retroflexus* L. Plants.

[42] Tseng T.-M., Shrestha S., McCurdy J.D., Wilson E., Sharma G. (2019). Target-site mutation and fitness cost of acetolactate synthase inhibitor-resistant annual bluegrass. Hortscience.

[43] Vercellino R.B., Hernández F., Pandolfo C.E., Cantamutto M., Presotto A. (2021). Ecological fitness cost associated with the AHAS Trp574Leu mutation in feral *Raphanus sativus*. Weed Res..

[44] Leon R.G., Dunne J.B., Gould F. (2021). The role of population and quantitative genetics and modern sequencing technologies to understand evolved herbicide resistance and weed fitness. Pest Manag. Sci..

